# Improved dementia screening for elderly with low education in South Korea using the Cognitive Impairment Screening Test (CIST)

**DOI:** 10.3389/fnins.2025.1599019

**Published:** 2025-05-14

**Authors:** Kyung Wook Kang, Gilsoon Park, Hyunsoo Kim, Soo Hyun Cho, Seong-Min Choi, Kang-Ho Choi, Hak-Loh Lee, Gwangsoon Shon, Byeong C. Kim, Hosung Kim

**Affiliations:** ^1^Department of Neurology, Chonnam National University Medical School and Hospital, Gwangju, Republic of Korea; ^2^USC Stevens Neuroimaging and Informatics Institute, Keck School of Medicine of USC, University of Southern California, Los Angeles, CA, United States; ^3^Gwangju Provincial Dementia Center, Gwangju, Republic of Korea

**Keywords:** cognition, cognitive assessment screening instrument, MMSE, CIST, low education

## Abstract

**Background:**

The Mini-Mental State Examination (MMSE) is the most widely used cognitive screening test worldwide; however, it often overdiagnoses older adults with low education levels. In contrast, the Cognitive Impairment Screening Test (CIST), developed by South Korea’s Ministry of Health and Welfare, may address this shortcoming. In this study, we compare the CIST and the Korean version of the MMSE (K-MMSE) in older adults with no formal education.

**Methods:**

We included 100 older adults (≥ 65 years)—27 with normal cognition (NC), 37 with mild cognitive impairment (MCI), and 36 with dementia (DM). All completed both the CIST and K-MMSE. First, we analyzed correlations between the CIST and K-MMSE. Next, we performed an analysis of covariance (ANCOVA), adjusting for age and sex, to compare group performance. Finally, classification performance was evaluated using receiver operating characteristic (ROC) curve analyses, examining the area under the curve (AUC) and other relevant metrics.

**Results:**

The CIST showed positive correlations with both the K-MMSE (*r* = 0.722) and the K-MMSE z-score (*r* = 0.625). ANCOVA revealed significant group differences (*p* < 0.001) for both measures. When distinguishing NC from MCI/DM, the CIST outperformed the K-MMSE, demonstrating a higher AUC (0.869 vs. 0.842) and F1-score (0.697 vs. 0.409).

**Conclusion:**

The CIST is a reliable and useful tool for assessing cognitive function, showing advantages over the K-MMSE in detecting cognitive decline among older adults without formal education. Further large-scale validation studies are warranted.

## 1 Introduction

Early diagnosis and timely intervention of cognitive impairment are the most effective strategies to enable personalized care. Specifically, personalized care can target the 12 modifiable risk factors for cognitive impairment identified by Livingston et al. (2020), reduces exposure to potentially inappropriate medications, and allows for the timely administration of emerging therapies for dementia-causing diseases such as Alzheimer’s disease (AD). Such therapies have been shown to the most effective when administered at earlier stages of the disease (Tzeng et al., 2024). So, national dementia policies are essential for early detection and management (Hampel et al., 2022), and the World Health Organization (WHO) also emphasizes early intervention to slow disease progression (World Health Organization [WHO], 2019). Many governments have adopted policies to manage dementia, exemplified by South Korea’s National Responsibility Policy for Dementia Care, which established a nationwide network of 256 Dementia Reassurance Centers (DRC) (Byeon et al., 2023; Lee, 2019).

Compared to many other countries, South Korea has made notable progress in dementia care by implementing robust policies, such as covering up to 90% of dementia-related medical expenses under its national health insurance system (Byeon et al., 2023; Choi et al., 2024; Hampel et al., 2022). The primary objective of these policies is the early diagnosis of cognitive decline, which is supported by including a dementia screening test. Globally, the Mini-Mental State Examination (MMSE) is recognized as the most widely used cognitive screening tool, designed to assess various cognitive domains, and available in multiple languages (Folstein et al., 1975; Owens et al., 2020). In South Korea, several screening tools adapted from the MMSE have been developed, among which the Korean-Mini Mental State Examination (K-MMSE) that retains most of the original MMSE items has become one of the most commonly utilized (Kang et al., 1997).

However, the MMSE may have limitations in diagnosing mild cognitive impairment (MCI) or early-stage dementia, particularly for individuals with low education levels, who still make up more than 20% of the global population (Kang et al., 1997; Baek et al., 2016; Devenney and Hodges, 2017; Statistica, 2024). This is because the MMSE’s tasks often require skills influenced by formal education, such as reading, writing, and arithmetic, which can lead to lower scores for those with limited schooling (Matallana et al., 2011). Consequently, individuals with low education or illiteracy may be misclassified as cognitively impaired when they are not (Devenney and Hodges, 2017). Although South Korea ranks relatively high in educational attainment among OECD countries—with 69.58% of adults aged 25 to 34 having received tertiary education (OECD, 2022)—a 2023 door-to-door survey of 10,078 elderly individuals revealed that 40.5% had six or fewer years of schooling, highlighting a notable generational disparity (MOHW, 2024). Similarly, a descriptive study of 18 OECD countries reported that education levels tend to decline with age (Murtin et al., 2022). Therefore, caution is required when interpreting K-MMSE results, as older adults, the primary targets of dementia screening tests, are more likely to have low education attainment.

Another challenge is that the MMSE is heavily influenced by language and cultural backgrounds, making it difficult for the Korean-adapted version, the K-MMSE, to accurately capture the intended meaning of certain items in the Korean context (Devenney and Hodges, 2017; Han et al., 2008; Shim et al., 2017). For example, the MMSE includes the English idiom “no ifs, ands, or buts,” which features repetitive vowel sounds. This was adapted in the K-MMSE as “seeing is believing,” (“baek-mun-yi-bul-yeo-il-gyen”), which not only changes the original meaning but also lacks the repetitive vowel sounds present in the English phrase. This adaptation fails to convey an equivalent meaning or phonetic pattern, highlighting a cultural and linguistic mismatch (Noroozian et al., 2014; Devenney and Hodges, 2017; Han et al., 2008; Shim et al., 2017). Moreover, previous Western studies have reported that literacy significantly influences financial capacity, subsequently affecting activities of daily living (Giannouli and Tsolaki, 2021). However, considering linguistic and cultural differences inherent in the Korean context, illiteracy may not necessarily be associated with financial incapacity, as previously reported in Western studies. Thus, it is crucial to recognize that the implications of low education levels in dementia screening tests within Korean culture may differ from those observed in Western studies using tools originally developed for Western populations.

Therefore, since 2021, The Ministry of Health and Welfare (MOHW) in South Korea has developed and started using the Cognitive Impairment Screening Test (CIST), which was designed with consideration of the country’s linguistic and cultural background, and includes more picture-based items compared to the K-MMSE. However, there has been no validation of the CIST in elderly individuals with low education. We aimed to validate the diagnostic utility of CIST compared to the K-MMSE in elderly individuals with no formal education. We also assessed the compatibility between CIST and K-MMSE. Through this systematic evaluation, we sought to demonstrate the advantages of using CIST as a screening tool at DRC, as part of South Korea’s national dementia care policy.

## 2 Materials and methods

### 2.1 Populations

This retrospective study was conducted from 2022 to 2023 among older adults (aged 65 years or above) with no formal education who visited DRCs in Gwangju City or the dementia clinic at Chonnam National University Hospital in South Korea. To assess participants’ cognitive status, trained neuropsychologists administered either the second edition of the Seoul Neuropsychological Screening Battery (SNSB-II) or the Korean version of the Consortium to Establish a Registry for Alzheimer’s Disease (CERAD-K), thereby differentiating normal cognition (NC), MCI, and dementia (DM). Based on these results, a neurologist made the final diagnoses. The diagnosis of MCI was based on Petersen’s criteria and required (1) cognitive test scores that are more than 1.5 standard deviation below age-, sex-, and education-adjusted norms in at least one cognitive area, (2) preserved ability to perform daily activities, and (3) no signs of dementia (Petersen, 2004). Dementia cases in this study comprised AD and subcortical vascular dementia (SVaD). Probable AD was diagnosed according to the National Institute on Aging and Alzheimer’s Association research criteria (McKhann et al., 2011), while SVaD was diagnosed when dementia coincided with prominent subcortical vascular features (Kim et al., 2014). Consequently, the analysis included 36 patients with DM (mean age: 84.3 5.6 years, female: 91.7%), 37 with MCI (mean age: 80.75.4, female: 83.8%), and 27 with NC (mean age: 78.13.2 years, female: 92.6%). All participants underwent the CIST and K-MMSE at the time of screening. This study was approved by the Institutional Review Board of Chonnam National University Hospital (IRB no. CNUH-2024-197), and all participants provided written informed consent.

### 2.2 CIST

The CIST was developed by South Korea’s MOHW as part of its national dementia care policy and has been used in DRCs since 2021 (National Institute of Dementia, 2021). Designed to reflect the specific circumstances and needs of South Korea, the CIST is administered as a face-to-face test. Comprising 13 items for a total of 30 points, the CIST takes about 10 min to administer and assesses global cognitive functions across six cognitive domains: orientation, memory, attention, visuospatial ability, language, and executive function (EF) (Supplementary Table 1).

### 2.3 K-MMSE

The K-MMSE was adapted from the original MMSE, developed by Folstein et al. (1975), to better reflect the cultural context of South Korea. Although several items were modified for this purpose, efforts were made to retain the original structure and content as much as possible (Kang et al., 1997). Consequently, the K-MMSE still shares the limitations of the original MMSE (Kang et al., 1997; Han et al., 2008). The K-MMSE evaluates multiple cognitive domains, including time orientation (5 points), spatial orientation (5 points), memory registration (3 points), attention and calculation (5 points), memory recall (3 points), language (8 points), and visuospatial construction (1 point) (Supplementary Table 1). The total score ranges from 0 to 30, and administration typically takes 5–10 min. We calculated z scores using the mean and standard deviation values from age- and education-adjusted norms (Han et al., 2008) and defined cognitive impairment as a z score of −1 or below.

### 2.4 Statistical analysis

Pearson’s correlation coefficients were calculated to examine the relationships between the total CIST score, CIST subdomain scores, K-MMSE total score, and K-MMSE z-scores.

To evaluate the ability of each screening method to differentiate cognitive performance across the three diagnostic groups—normal cognition (NC), mild cognitive impairment (MCI), and dementia (DM)—we conducted analysis of covariance (ANCOVA) to compare CIST scores, K-MMSE scores, and K-MMSE z-scores among the groups. Age and sex were included as covariates in the ANCOVA models. Post-hoc analyses were conducted using two-sample *t*-tests to compare group means.

To evaluate the discriminatory power of each screening test, Receiver Operating Characteristic (ROC) curve analysis was performed. area under the curve (AUC) values were calculated for the following group comparisons: NC vs. MCI/DM, NC vs. MCI, NC vs. DM, and MCI vs. DM. For the NC vs. MCI/DM comparison, additional performance metrics were calculated, including F1-score, sensitivity, specificity, and accuracy, to provide a more comprehensive evaluation. The F1-score is the harmonic mean of sensitivity and specificity, providing a balanced measure of a test’s accuracy that considers both false positives and false negatives. A higher F1-score indicates better overall performance.

To assess the discriminatory power of cognitive subdomains within the CIST, ROC analyses were conducted separately for each subdomain (Orientation, Attention, Visuospatial function, EF, Memory, Language function), and AUC values were calculated as above.

All statistical analyses were performed using Matlab 2022a,^1^ and statistical significance was set at *p* < 0.05.

## 3 Results

### 3.1 Correlations between CIST total score, CIST subdomains, K-MMSE total score, and K-MMSE z-score

Table 1 shows the correlation coefficients among the CIST total score, CIST subdomains, K-MMSE total score, and K-MMSE z-scores. All correlations were statistically significant (*p* < 0.005). The CIST total score showed strong positive correlations with both the K-MMSE (*r* = 0.722) and K-MMSE z-score (*r* = 0.625). Among the CIST subdomains, Orientation (*r* = 0.599) and EF (*r* = 0.581) exhibited high correlations with the K-MMSE total score. For the K-MMSE z-score, EF (*r* = 0.506) and Orientation (*r* = 0.505) also showed relatively high correlations.

**TABLE 1:** Correlation coefficients (CCs) among CIST total score, CIST subdomains, K-MMSE total score, and K-MMSE z-score.

Cognitive domains of CIST	K-MMSE		K-MMSE (z-score)	
	**CC**	***P*-value**	**CC**	***p*-value**
Orientation	0.599	< 0.0001	0.505	< 0.0001
Memory	0.421	< 0.0001	0.352	0.0003
Attention	0.331	0.0008	0.312	0.0016
Visuospatial function	0.373	0.0001	0.371	0.0001
Language function	0.524	< 0.0001	0.436	< 0.0001
Executive function	0.581	< 0.0001	0.506	< 0.0001
CIST total score	0.722	< 0.0001	0.625	< 0.0001

CIST, Cognitive Impairment Screening Test; K-MMSE, Korean Mini-Mental State Examination.

### 3.2 Group differences in cognitive performance

ANCOVA revealed significant differences among the diagnostic groups for all three cognitive measures: CIST (*p* < 0.001), K-MMSE (*p* < 0.001), and K-MMSE z-score (*p* < 0.001) (Table 2A). Post-hoc *t*-tests demonstrated a consistent pattern of decreasing scores across groups (NC > MCI > DM) for all measures (Table 2A).

**TABLE 2:** Cognitive performance across diagnostic groups [normal control (NC), mild cognitive impairment (MCI), and dementia (DM)] for each screening score. Mean scores, standard deviations, and analysis of covariance (ANCOVA) results. Post-hoc analyses were performed using two-sample *t*-tests.

Scores	NC (*n* = 27)	MCI (*n* = 37)	DM (*n* = 36)	Post-hoc
CIST, mean ± SD	16.6 ± 4.1	12.2 ± 3.3	7.8 (3.9)	a > b > c
K-MMSE, mean ± SD	21.0 (3.4)	18.0 (3.7)	10.6 (5.3)	a > b > c
K-MMSE (z-score), mean ± SD	−0.36 (1.19)	−1.06 (1.06)	−2.62 (1.23)	a > b > c

*All ANCOVA tests had *p* < 0.001; a, NC; b, MCI; c, DM.

**TABLE 3:** Between-group comparisons using two-sample *t*-tests (*t*-values).

*T*-values	NC vs. MCI/DM	NC vs. MCI	NC vs. DM	MCI vs. DM
CIST	6.94	4.73	8.69	5.30
K-MMSE	5.53	3.27	8.97	6.99
K-MMSE (z-score)	4.89	2.48	7.32	5.81

*All *t*-tests had *p* < 0.001 except NC vs. MCI on K-MMSE (z-score) (*p* < 0.05). CIST, Cognitive Impairment Screening Test; K-MMSE, Korean Mini-Mental State Examination; SD, standard deviation.

In the Table 2B, CIST exhibited higher *t*-values than the K-MMSE in differentiating NC from MCI (4.73 vs. 3.27) and NC from any abnormal state (6.94 vs. 5.53), while all comparisons were significant at *p* < 0.001 except for the comparison of NC vs. MCI using K-MMSE (z-score). These results, especially high *t*-values of CIST suggest its superior discriminatory power for early detection.

### 3.3 Discriminatory power of CIST and K-MMSE

**FIGURE 1:**
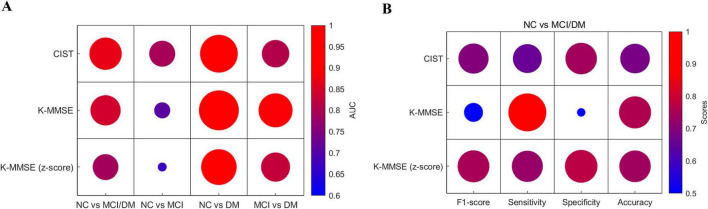
Discriminatory power of the CIST, K-MMSE, and K-MMSE z-score for cognitive impairment screening. **(A)** Area under the receiver operating characteristics curves (AUC) results about each group comparison. AUC values are as follows: for normal control (NC) vs. mild cognitive impairment (MCI)/dementia (DM): CIST = 0.8691, K-MMSE = 0.8422, K-MMSE z-score = 0.7900. For NC vs. MCI: CIST = 0.7928, K-MMSE = 0.7067, K-MMSE z-score = 0.6697. For NC vs. DM: CIST = 0.9475; K-MMSE = 0.9815; K-MMSE z-score = 0.9136. For MCI vs. DM: CIST = 0.8093; K-MMSE = 0.8915; K-MMSE z-score = 0.8266. the CIST had higher AUC values than the K-MMSE and K-MMSE z-scores for distinguishing NC from MCI/DM and NC from MCI. Conversely, the K-MMSE demonstrated the highest AUC for discriminating between NC and DM and between MCI and DM. **(B)** Performance metrics for classification of NC vs. MCI/DM. CIST: F1-score = 0.6967, Sensitivity = 0.7397, Specificity = 0.7407, Accuracy = 0.7400. K-MMSE: F1-score = 0.4094, Sensitivity = 0.9726, Specificity = 0.2593, Accuracy = 0.7900. K-MMSE z-score: F1-score = 0.7510, Sensitivity = 0.7123, Specificity = 0.7778, Accuracy = 0.7300. the K-MMSE z-score achieved the highest F1 score and the CIST achieved the second highest F1 score. Although the K-MMSE had a high sensitivity, it had a significantly low specificity, resulting in the lowest F1 score due to a high false positive rate.

ROC curve analyses (Figure 1A) showed that the CIST had higher AUC values than the K-MMSE and K-MMSE z-scores for distinguishing NC from MCI/DM (CIST: 0.8691; K-MMSE: 0.8422; K-MMSE z-score: 0.7900) and NC from MCI (CIST: 0.7928; K-MMSE: 0.7067; K-MMSE z-score: 0.6697). Conversely, the K-MMSE demonstrated the highest AUC for discriminating between NC and DM (CIST: 0.9475; K-MMSE: 0.9815; K-MMSE z-score: 0.9136) and between MCI and DM (CIST: 0.8093; K-MMSE: 0.8915; K-MMSE z-score: 0.8266).

Further examination of the NC vs. MCI/DM classification (Figure 1B) revealed that the K-MMSE z-score achieved the highest F1 score (0.7510) and the CIST achieved the second highest F1 score (0.6967). Although the K-MMSE had a high sensitivity (0.9726), it had a significantly low specificity (0.2593), resulting in the lowest F1 score (0.4094) due to a high false positive rate.

### 3.4 Subdomain discriminatory power of CIST

Table 3 presents the AUC values for each CIST subdomain across the different group comparisons. Orientation and EF consistently demonstrated the highest AUC values. Orientation showed the highest AUC for NC vs. DM (0.9367) and MCI vs. DM (0.8960), and the second-highest for NC vs. MCI/DM (0.7765). EF exhibited the highest AUC for NC vs. MCI/DM (0.8262) and NC vs. MCI (0.7653), and the second-highest for NC vs. DM (0.8889).

**TABLE 4:** Area under the receiver operating characteristic curve (AUC) values for CIST subdomains in discriminating between diagnostic groups.

AUC	NC vs. MCI/DM	NC vs. MCI	NC vs. DM	MCI vs. DM
Orientation	0.7765	0.6206	**0.9367**	**0.8960**
Memory	0.6953	0.6517	0.7402	0.6284
Attention	0.5715	0.5105	0.6343	0.6160
Visuospatial function	0.6938	0.7062	0.6811	0.4895
Language function	0.6651	0.5706	0.7623	0.7035
Executive function	**0.8262**	**0.7653**	0.8889	0.6948

Bold, highest AUC. CIST, Cognitive Impairment Screening Test; NC, normal control; MCI, mild cognitive impairment; DM, dementia.

## 4 Discussion

To our knowledge, this is the first study to compare the discriminatory power of the CIST and the K-MMSE among older adults with no formal education in a community setting. We found that the CIST was highly compatible with both the K-MMSE and K-MMSE z scores, suggesting that the CIST can be a useful tool for assessing cognitive function of older adults with low levels of education. Among the six subdomains of the CIST, orientation showed the highest compatibility with the K-MMSE (CC = 0.599, *p* < 0.0001), likely because it accounts for a large portion (33.3%) of the total K-MMSE score (Kang et al., 1997).

In addition, both the CIST and K-MMSE scores showed a statistically significant and progressive decline from NC to MCI and DM. Notably, the CIST yielded higher *t*-values than the K-MMSE when differentiating NC from MCI (4.73 vs. 3.27) and when distinguishing NC from any abnormal state (6.94 vs. 5.53), suggesting that it may be a more suitable screening tool for early detection of cognitive decline. When the CIST was classified as NC or abnormal (MCI or DM) based on MOHW normative data stratified by age and education (National Institute of Dementia, 2021), it demonstrated greater discriminating power than the K-MMSE < 24 cutoff (F1 score: CIST = 0.6967 vs. K-MMSE = 0.4094) in identifying individuals who require comprehensive neuropsychological testing—including the assessment of various cognitive domain. However, applying the K-MMSE z score < −1 criterion produced the highest F1-score. In ROC analyses for differentiating NC from abnormal groups, the CIST also showed better performance than the K-MMSE, although the difference did not reach statistical significance (AUC: CIST = 0.8691 vs. K-MMSE = 0.84221). We believe these findings may be attributed to the CIST’s inclusion of an EF domain, which the K-MMSE does not measure (Folstein et al., 1975; National Institute of Dementia, 2021; Kang et al., 1997). As shown in Table 3, EF also exhibited the highest AUC among various cognitive domains for distinguishing NC from MCI and DM or from MCI alone (AUC: NC vs. MCI/DM = 0.8262, NC vs. MCI = 0.7653).

Historically, AD was recognized primarily for its early and prominent memory deficits, with EF believed to remain largely intact. However, emerging evidence indicates that even patients with mild AD can exhibit significant EF impairments (Grober et al., 2008; Junquera et al., 2020; Dickerson and Wolk, 2011). For example, a volunteer-based longitudinal cohort study that followed participants for up to 15 years before AD diagnosis found that EF performance declines accelerated 2–3 years prior to diagnosis (Grober et al., 2008). Another study reported that EF impairment in very mild AD is not uncommon and is associated with more rapid disease progression compared to a typical amnestic phenotype (Dickerson and Wolk, 2011). Similarly, a study assessing neuropsychological performance at baseline and after one year in patients with MCI found that impaired EF significantly predicts conversion to dementia in Alzheimer’s clinical syndrome one year later (Junquera et al., 2020).

Higher-level EF, which is essential for daily functioning as well as physical and mental health, has been shown to deteriorate in older adults with MCI compared to healthy older adults (Corbo and Casagrande, 2022). Furthermore, as age increases, not only does the prevalence of AD rise, but the prevalence of non-AD dementias—such as vascular or frontotemporal dementia—also increases (O’Brien and Thomas, 2015). Because these forms of dementia frequently affect EF, it is important to include EF assessments in MCI screening tests for older individuals. However, the K-MMSE is known to be relatively insensitive to EF impairments (Kang et al., 1997; Devenney and Hodges, 2017). Consequently, relying solely on this measure may lead to an underestimation of executive dysfunction, which can emerge even in the early stages of MCI (Dickerson and Wolk, 2011; Grober et al., 2008; Junquera et al., 2020; Devenney and Hodges, 2017), ultimately delaying appropriate treatment for individuals with MCI (Devenney and Hodges, 2017). In contrast, the CIST assesses EF through visual reasoning, verbal reasoning, and verbal fluency tasks, each worth up to 2 points, for a total of 6 points (Supplementary Table 1). At a minimum, community-based dementia screening tests targeting older adults should include an EF assessment. In our study, the CIST demonstrated its potential as a dementia screening tool in community DRCs (Lee, 2019). However, further large-scale research, including a normative sample, is necessary to validate its effectiveness.

Furthermore, the MMSE has significant additional limitations due to ceiling and floor effects across different education levels. Specifically, highly educated individuals with MCI often yield false negatives, while older or illiterate individuals more prone to false positives (Noroozian et al., 2014). Because the K-MMSE allocates a larger proportion of its items to language function—which is strongly influenced by education (Noroozian et al., 2014)—it is more susceptible to educational background than the CIST. Notably, a meta-analysis of studies using the MMSE or its adapted versions as cognitive screening tools reported that the difference in dementia screening scores between literate and illiterate older adults was even greater among individuals without cognitive impairment (Maher and Calia, 2021). This finding suggests that the MMSE may mistakenly classify cognitively normal older adults with illiteracy as having cognitive decline, highlighting its floor effect in this population. Given that the K-MMSE includes many tasks involving language and calculation—domains that can be particularly challenging for older adults with low formal education (Pellicer-Espinosa and Díaz-Orueta, 2022)—caution is warranted when interpreting K-MMSE results in this population. Indeed, our study confirmed that the false positive rate of the K-MMSE for detecting abnormal states beyond MCI was notably higher than that of the CIST (0.74 vs. 0.26, respectively).

The MMSE was originally designed as a quick bedside screening tool for inpatients with dementia or other psychiatric disorders (Folstein et al., 1975); consequently, it has inherent limitations. Numerous studies have shown that both the original MMSE and its adaptations rely heavily on reading, writing, and arithmetic skills, which systematically lower the scores of older adults with low education levels (Devenney and Hodges, 2017; Maher and Calia, 2021; Noroozian et al., 2014). To reduce this bias, Xu et al. (2003) developed the Chinese Adapted MMSE (CAMSE), modifying several items to minimize literacy dependency and to make it more suitable for Chinese culture. However, even after these revisions, the serial sevens subtraction task still showed a significant difference between literate and illiterate individuals in the non-demented group (Xu et al., 2003). This residual bias may contribute to the overdiagnosis of cognitive impairment in cognitively normal older adults with little or no formal education. Therefore, moving beyond the MMSE and developing cognitive screening tools that are valid for all individuals regardless of their education level, literacy ability, or cultural background is essential.

Our study has several limitations. First, it was a retrospective study with a relatively small sample size. We suggest that future studies replicate these findings using nationwide data, given that the CIST is currently utilized as a dementia screening test in community DRCs across South Korea. Second, we were unable to control the potential effects of medication on each participant’s cognitive function. Third, although 40.5% of South Koreans aged 65 and older have six years or less of education, only 12.3% have received no formal education. Since our study focused exclusively on older adults with no formal education who visited our local DRC during the study period, our sample may not be representative of the broader elderly population in South Korea. Fourth, our study specifically compared the CIST with K-MMSE among elderly individuals with no formal education, which can limit the generalizability of our results. Further validation with participants from diverse educational and cultural backgrounds is necessary to clarify the advantages of the CIST identified in this study. Finally, although the composite EF score showed robust discriminatory accuracy, task-level data were unavailable for item-level AUC analyses. Future studies should report task-level EF metrics to facilitate replication and enable more detailed interpretation of CIST performance.

## 5 Conclusion

In conclusion, the findings of this study show that the CIST meets these criteria by showing advantages over the K-MMSE in detecting cognitive decline among older adults without formal education. Consequently, it may serve as a more suitable screening tool under the National Responsibility Policy for Dementia Care in South Korea. Notably, the CIST incorporates a greater number of picture-based items compared to the K-MMSE, which is particularly useful for populations with limited formal education. Although the K-MMSE z score, derived from previously published normative data, showed higher F1 scores for distinguishing abnormal groups from NC in this study, we anticipate that this discrepancy will be resolved once normative data for the CIST, stratified by age and education, become available.

## Data Availability

The raw data supporting the conclusions of this article will be made available by the authors, upon request.

## Footnotes

1
https://www.mathworks.com/
