# Supplementary material for: Improved dementia screening for elderly with low education in South Korea using the Cognitive Impairment Screening Test (CIST)
Source: Front Neurosci. 2025 May 14;19:1599019. doi: 10.3389/fnins.2025.1599019 (PMC12116617; doi:10.3389/fnins.2025.1599019)
Supplement: Supplementary file 1 [file Table_1.docx]

**Supplementary Material**

**Supplementary Table 1.** Comparison of cognitive domains and items between the Cognitive Impairment Screening Test (CIST) and the Korean Mini-Mental State Examination (K-MMSE).

|  |  | **CIST** | | **K-MMSE** | |
| --- | --- | --- | --- | --- | --- |
|  |  | **Inclusion** | **Score** | **Inclusion** | **Score** |
| **Orientation** | Time | ○ | 4 | ○ | 5 |
|  | Place | ○ | 1 | ○ | 5 |
| **Memory** | Registration | x | - | ○ | 3 |
|  | Recall | ○ | 10 | ○ | 3 |
|  | Recognition | ○ |  | x | - |
| **Attention** | Digit span forward | ○ | 2 | x | - |
|  | Word span backward | ○ | 1 | x | - |
|  | Calculation | x | - | ○ | 5 |
| **Visuospatial function** | | ○ | 2 | ○ | 1 |
| **Language function** | | ○ | 4 | ○ | 8 |
| **Executive function** | | ○ | 6 | x | - |
| **Total Score** | |  | 30 |  | 30 |
